# Psychosocial burdens in palliative care – a longitudinal cohort study in nursing homes and impacts of the COVID-19 pandemic

**DOI:** 10.1186/s12904-023-01292-4

**Published:** 2023-10-28

**Authors:** Anna Bußmann, Natalie Pomorin

**Affiliations:** 1 Essener Forschungsinstitut für Medizinmanagement GmbH, Essen, Germany; 2 FOM Hochschule für Oekonomie & Management gemeinnützige Gesellschaft mbH, Düsseldorf, Germany

**Keywords:** Palliative care, Terminal care, Hospice and palliative care nursing, Nursing homes, Workload, COVID-19, SARS-CoV-2

## Abstract

**Background:**

In Germany, palliative care in nursing homes is becoming increasingly important. Simultaneously, nursing homes are particularly affected by the COVID-19 pandemic due to their vulnerable residents leading to increased burdens for nursing staff. Although a separate unit for palliative care may not be present in nursing homes as it is in, e.g., hospitals, palliative care occupies a large portion of the workday in nursing homes. As no study addressing this topic could be found, this study focused on the research questions of how the psychosocial burdens faced by nursing staff in palliative care have been affected by the COVID-19 pandemic and how those burdens differ from the psychosocial burdens encountered in general care.

**Methods:**

Basen on a longitudinal cohort study design, a total of 113 nurses, nursing assistants and caregivers drawn from two nursing homes in North Rhine-Westphalia, Germany, were surveyed pre-pandemic in 2019 and during the pandemic in 2022 using the Copenhagen Psychosocial Questionnaire (COPSOQ) III. Data were examined descriptively following the standardised COPSOQ procedure. Additionally, chi-squared test was conducted to investigate the homogeneity between the groups. Mean differences (MD) were provided and Cohen’s d was calculated to evaluate relevant differences in psychosocial burdens between 2019 and 2022. In a second step, t-tests were performed to test statistical significance.

**Results:**

Relevant positive changes could be identified in ‘Quantitative demands’ (d = 0.321; MD = 5.9), ‘Influence at work’ (d = 0.244; MD = 5.4), ‘Job insecurity’ (d = 0.321; MD = 6.5), ‘Insecurity over working conditions’ (d = 0.296; MD = 6.8), ‘Burnout symptoms related to residents’ (d = 0.201; MD = 3.8), ‘Degrees of freedom’ (d = 0.455; MD = 9.6) and ‘Presenteeism’ (d = 0.425; MD = 11.8). Relevant negative changes were found in ‘Dissolution’ (d = 0.217; MD = 5.4; i.e., setting boundaries between work and private life), ‘Role conflicts’ (d = 0.282; MD = 5.5), ‘Role clarity’ (d = 0.251; MD = 3.3) and ‘Burnout symptoms related to relatives’ (d = 0.318; MD = 6.0). Relevant changes that were statistically significant according to the t-test could be identified in ‘Degrees of freedom’ (t-value=-2.40; p = 0.018) and ‘Presenteeism’ (t-value = 2.26; p = 0.026). Responses to questions concerning nursing homes’ handling of the COVID-19 pandemic exhibited a mean score of 68.2 for ‘Organisation/communication’ and a mean score of 78.1 concerning ‘Operational measures and overall assessment’ during the COVID-19 pandemic.

**Conclusions:**

Besides negative changes during the COVID-19 pandemic, some categories showed more positive results. The burdens of palliative care in nursing homes may be perceived differently than those of general care in nursing homes. Furthermore, the results indicate that perceptions of challenges in palliative care in nursing homes during the pandemic seem to be highly dependent on organisational working conditions and support that can strengthen the individual resources and resilience of the staff.

**Supplementary Information:**

The online version contains supplementary material available at 10.1186/s12904-023-01292-4.

## Background

Palliative care is defined as the holistic care of patients, especially patients who are near the end of life and who experience serious health-related suffering due to severe illness [1]. Palliative care aims to improve the quality of life of patients and their families by considering individual needs and by relieving social, spiritual, physical, and psychological forms of pain and suffering [1].

In nursing homes, palliative care is becoming increasingly important. As in many Western countries, the growth of the ageing and multimorbid population in Germany has led to increases in the number of people in need of nursing care over the past years [2]. Furthermore, demographic and epidemiological changes have caused shifts in the location of death towards nursing homes [3]. Nursing homes play an important role as the location of death, accounting for 22.5% of all deaths according to a study based on death register data in Germany from 2018 to 2021 [4].

Palliative care services in Germany can be divided into general and specialised services and are offered in either inpatient or outpatient settings. For patients who suffer from particularly complex symptoms and needs, German statutory health insurance covers specialised outpatient palliative care (SAPV), which is provided by multiprofessional teams at the patient’s residence, including nursing homes [5]. In general outpatient palliative care (AAPV), the medical aspect is typically addressed by general practitioners who also care for nursing home residents. General palliative care in nursing homes is by law included into care practice (§ 28 Abs. 4 SGB XI) without any additional funding or requirements for palliative care-specific qualifications. It accounts for approximately 90% of all palliative cases [6] and is the focus of the present study. In nursing homes, palliative care is not usually provided in separate care units as in the case of, e.g., hospitals or hospices. On average, nursing staff in nursing homes spend 20% of their working time providing general palliative care [7].

Registered nurses, who are responsible for most tasks and nursing activities involved in palliative care in nursing homes, have completed three years of training, while nursing assistants are responsible for providing assistance in palliative care and have usually completed one year of professional training. Caregivers are not involved in body-related care [8]. They assist and activate residents according to their needs with the goal of improving their physical and psychological wellbeing, e.g., by providing additional attention and company, more interactions with other people and more participation in life [8].

The demands on nursing staff involved in palliative care are high. Palliative care is associated with severe physical, psychological, and social stress [9]. Care needs are exacerbated due to the complexity and variety of symptoms suffered at the end of life and thus require more resources [9]. Moreover, organisational factors in nursing homes, such as long hours on duty, staff shortages, and irregular shift schedules alongside increasing working demands on nursing staff, are perceived as stressful [10, 11]. Time-consuming documentation, conflicts with relatives of dying patients and the daily exposure to grief, death, and sorrow act as further emotional stressors [7, 9–11]. In a cross-sectional study concerning working conditions in palliative care conducted in German nursing homes, more than half (55.5%) of the 346 participating nurses perceived palliative care as highly emotionally challenging, and encounters with dying residents accounted as a burden for 80% of the nurses [7].

During the coronavirus disease (COVID)-19 pandemic, German nursing homes, like other nursing homes worldwide encountered new challenges, which have been investigated in numerous studies [12–14]. Nursing home residents represent a very vulnerable group that exhibits a high risk of suffering and death due to severe cases of COVID-19 [15]. Furthermore, outbreaks in nursing homes are common due to the rapid spread of infections in shared accommodations [14]. As of November 2022, almost 350 thousand COVID-19 cases had been reported among nursing home residents in Germany [16], and approximately half of all COVID-19 deaths were among nursing home residents [14]. As a result, nursing homes have been greatly impacted by the pandemic, leading to a significant increase in the physical and mental workloads [14, 17–21]. High rates of sick leave and staff shortages, daily readjustments of work processes, limited possibilities to exchange work information with colleagues, the increased mortality rate of residents with COVID-19 and fears of becoming infected and infecting residents are perceived burdens [14, 17–21].

COVID-19 also causes further emotional stress and intensified workloads in the context of palliative care [22–24]. In particular, conflicts between infection control regulations and the provision of adequate palliative care in terms of personal, psychosocial, and emotional support are perceived as burdens in palliative care [23]. Furthermore, COVID-19 leads to contact restrictions, the reorganisation of palliative care, additional tasks for staff and new forms of palliative care delivery, which lead to work overload, distress, and burnout in addition to affecting health and well-being [23, 24].

Although some studies have investigated the impacts of the pandemic on palliative care in Germany [23, 25–30] or on the workloads faced by nursing home staff [17, 21], no study that specifically addressed the psychosocial burdens of palliative care in nursing staff working in German nursing homes and the impact of the COVID-19 pandemic could be found. Therefore, this research focused on the questions of how the psychosocial burdens of palliative care in nursing homes have been affected by the COVID-19 pandemic and how these psychosocial burdens differ from those of general care. As palliative care in nursing homes is often overlooked but nevertheless plays an important role in everyday care practice, this study aims to contribute to this field of research by providing further insights into the psychosocial burdens of palliative care in nursing homes.

## Methods

### Study design

Data collection was performed as part of the study ‘Hospice and palliative care in nursing homes’, a three-year publicly funded study aimed at improving hospice care and palliative care in nursing homes. Data were collected from nursing staff (nurses, nursing assistants and caregivers) using a longitudinal cohort study design featuring a quantitative research approach. Nursing staff drawn from two nursing homes in North Rhine-Westphalia, Germany were surveyed at two time points, and each survey period covered 6 weeks. The first survey period took place from 03.10.2019–14.11.2019. The second survey was conducted 2.5 years after the first survey period, i.e., from 25.04.2022-03.06.2022, thus taking place during the COVID-19 pandemic. In March 2020, the federal and state governments agreed on guidelines to combat the spread of the coronavirus, which included restrictions on social contact and lockdowns [31], which varied among the federal states over time. Two years after the first nationwide COVID-19 guidelines were implemented, the time period for the second survey was chosen, which took place one month after coronavirus restrictions regarding contact restrictions had been greatly relaxed in the state of North Rhine-Westphalia in line with a new coronavirus protection regulation and changes to the German Infection Protection Act [32]. The obligation to wear face masks, COVID-19 testing plans, and compliance with required hygiene concepts remained unaffected by this relaxation and were maintained. In both survey periods, a reminder was sent to participants two weeks before the end of the period.

### Study participants

Nursing staff drawn from two nursing homes associated with a private provider were surveyed. The criteria for inclusion were that the institutions should be general nursing homes, regularly provide palliative care, employ palliative care nurses, be located in North Rhine-Westphalia, Germany, and be willing to participate in the study. Nursing homes with certain specifications (e.g., nursing homes aimed only at residents with dementia) were excluded. The number of eligible study participants was determined a priori. Nurses, nursing assistants and caregivers were eligible for participation. Although both surveys were conducted in the same two nursing homes, the samples were considered to be independent. This assumption was made due to staff turnover and for reasons of anonymity, as participants were not tracked, although the possibility that certain participants took part in both surveys cannot be excluded. The recruitment process involved writing a letter to potential study participants. In this letter, information about the study’s objectives and the voluntary nature of the survey were provided. Furthermore, the procedure of the COPSOQ survey was explained, and anonymity was assured. Informed consent was given by participation in the survey. Participants were paid one hour’s worth of overtime compensation for completing the survey. To guarantee the anonymity of the participating staff, this extra pay was provided to all staff members.

### Questionnaire

The paper-based surveys were based on the third version of the German Copenhagen Psychosocial Questionnaire (COPSOQ). The COPSOQ is a reliable instrument that has been widely used internationally to assess psychosocial working conditions within diverse occupational fields [33]. Its scales are organised based on model of cause and effects, including aspects of several important psychosocial theories, such as the demand-control-support model, the effort-reward imbalance model and the job demands-resources model [34]. The German version III of this questionnaire was validated in 2021 and has exhibited good or even very good reliability (Cronbach’s α ≥ 0.7) with regard to 28 of its 31 scales and satisfying or even good homogeneity (intraclass-coefficient ICC ≥ 0.5) for 24 of these 31 scales [34]. The 31 scales of the questionnaire include a total of 84 items pertaining to psychosocial work stress and its effects. Sociodemographic data included age, sex, profession, working hours, management position (personnel responsibility, e.g., as a ward manager), and palliative care-specific qualifications (e.g., have completed a specialised 160-hour nursing training programme in palliative care or a 40-hour basic module for palliative care for nursing assistants and caregivers, both of which are stipulated by the Deutsche Gesellschaft für Palliativmedizin e.V.). In both surveys, an extension of the COPSOQ standard version was employed, which included four validated sector-specific modules of the COPSOQ. Two of these modules referred to the psychosocial impact of dealing with residents receiving palliative care, while the other two modules referred to the psychosocial impact of contact with relatives. Furthermore, two standardised COPSOQ modules pertaining to COVID-19 were added in the 2022 survey, resulting in 35 scales including a total of 139 items in the 2019 survey and 37 scales including a total of 141 items in the 2022 survey. Most of the items were scored on a five-point Likert-type scale. For example, with regard to the item ‘Is your work emotionally demanding?’, responses included ‘to a very large extent’, ‘to a large extent’, ‘somewhat’, ‘to a small extent’, ‘to a very small extent’. The question concerning general health was an exception and was scored on a ten-level scale ranging from the worst to the best conceivable state of health [34]. For questions used to characterise the study population, categorial responses were employed (Table 1). Further information about the conceptual basis of COPSOQ III can be found online. Moreover, the measures used in the questionnaire are publicly accessible on the COPSOQ website.

### Analyses

According to the standardized COPSOQ procedure, the paper-based questionnaire returns were sent by post to the independent national COPSOQ-Institute Freiburg Research Centre for Occupational Sciences (FFAW) to ensure anonymity and validity. Data were digitalised, validated, and aggregated, and scale values were presented as mean values. Therefore, in line with the standardised validated COPSOQ procedure, the scales were subsequently transformed into point values ranging from 0 (the minimum, e.g., ‘does not apply at all’) to 100 (the maximum, e.g., ‘fully applies’) [34].

Subsequently, the paper-based questionnaires were disposed of by the COPSOQ-Institute FFAW. Questionnaires with missing items were still included in the analyses if at least half of the relevant items were not missing and if they featured a minimum of five responses per item. Possible bias that could arise from missing data was not addressed. To describe the data, descriptive statistics (absolute and relative frequencies, mean values, and standard deviations) were used by the COPSOQ-Institute FFAW.

As both COPSOQ-surveys were administered within the same nursing homes but the participants may have varied between the 2019 and 2022 samples, chi-square test of homogeneity was performed to determine whether the two survey groups differed significantly and to address possible selection bias.

To evaluate the differences in psychosocial burdens between 2019 and 2022, Cohen’s d was used to calculate effect sizes. A Cohen’s d of ≥ 0.2 corresponds to a small, ≥ 0.5 corresponds to a medium and ≥ 0.8 to a large effect [35]. Additionally, in order to establish comparability for other COPSOQ-studies, mean differences (MD) were provided, as the approach in which a defined mean difference of at least 5 points is often found in COPSOQ studies to classify changes as relevant [36–38]. In a second step, t-tests were conducted to test the statistical significance of the effect sizes based on Cohen’s d previously identified as relevant. As several COPSOQ scales exhibited heterogeneity of variances (Hartley’s Fmax tests (95%-CI, α = 0.05), see ‘supplementary material’), Welch’s tests were performed (95%-CI, α = 0.05) since this approach has been recommended for unequal variances and still performs well when variances are equal [39]. Additional sensitivity analyses were not conducted. All analyses were performed using the software IBM SPSS Statistics 25 and Microsoft Excel Version 2211.

## Results

### Study population

**Fig. 1:**
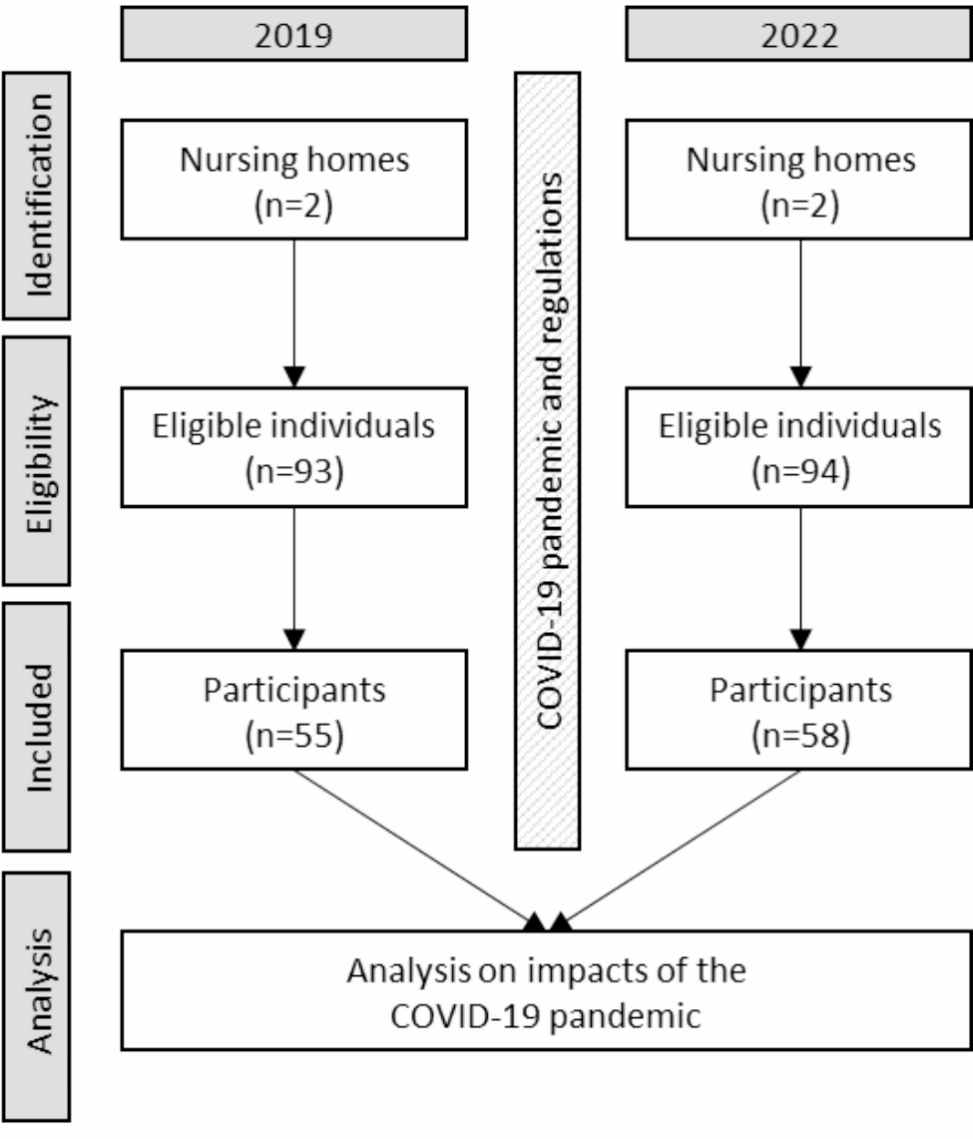
Flow chart

Samples from 93 individuals in 2019 and 94 individuals in 2022 were identified by the management of the two nursing homes as were eligible for participation and were thus potential recruits. In 2019, 55 of these potential recruits participated in the study, while in 2022, 58 potential recruits were willing to do so, resulting in good participation rates of 59% and 62%, respectively. As participation was voluntary and recruits were not required to state any reason for non-participation, 38 (in 2019) and 36 (in 2022) eligible individuals chose not to participate in the study. A flow chart of the process is provided in Fig. 1.

The characteristics of the study population were similar, as shown in Table 1, and no significant heterogeneity between 2019 and 2022 was indicated by the chi-squared test (significance level p < 0.05).

Most participants were between 45 and 54 years old. Approximately one-fifth of the respondents were 55 years or older. In 2019, the smallest group of participants was between 35 and 44 years old (16%), while in 2022, the smallest group was 34 years old or younger. Three quarters of the participants in both years were women, and approximately half of the respondents were nurses. Caregivers accounted for 25% of the total. In 2019, 16% of the participants had a leadership position, while in 2022, this figure was 26%. In both survey periods, more than 60% of the respondents did not have a full-time position. The proportion of nursing staff with relevant palliative-specific qualifications could not be presented, as some categories did not reach the minimum value for evaluation in 2019.

**Table 1:** Characteristics of the study population

Category	2019 n (%)	2022 n (%)	p-value^1^
**Participants**	55 (59)	58 (62)	
**Age grouped**			0.526
< 34	13 (23.6)	11 (19.0)	
35–44	9 (16.4)	16 (27.6)	
45–54	20 (36.4)	18 (31.0)	
> 55	12 (21.8)	13 (22.4)	
Missing	1 (1.8)	0 (0.0)	
**Sex**			0.432
Female	42 (76.4)	44 (75.9)	
Male	10 (18.2)	13 (22.4)	
Other/missing	3 (5.5)	1 (1.7)	
**Professions**			0.522
Registered nurse	25 (45.5)	31 (53.4)	
Nursing assistant	15 (27.3)	12 (20.7)	
Caregiver	14 (24.5)	15(25.9)	
Missing	1 (1.8)	0 (0.0)	
**Management position** ^**2**^			0.314
Yes	9 (16.4)	15 (25.9)	
No	38 (69.1)	33 (56.9)	
Missing	8 (14.5)	10 (17.2)	
**Full-time position**			0.836
Yes	18 (32.7)	20 (34.5)	
No	34 (61.8)	35 (60.3)	
Missing	3 (5.5)	3 (5.2)	

^1^ p-values based on chi-squared tests; ^2^ positions with responsibility over personnel, e.g., as a ward manager

### Changes in psychosocial burdens

Relevant changes could be identified across eleven categories given the threshold of a minimum effect size of 0.2 [35].

Positive changes in workload could be identified in the context of ‘Quantitative demands’ (d = 0.321; MD = 5.9), which were perceived as less burdensome in 2022 compared to 2019. Furthermore, participants felt that they had more ‘Influence at work’ in 2022 than in 2019 with a small effect size of 0.244 and a mean difference of 5.4. Additionally, ‘Job insecurity’ and ‘Insecurity over working conditions’ improved from 2019 to 2022, exhibiting small effect sizes of 0.321 (MD = 6.5) and 0.296 (MD = 6.8), respectively. ‘Burnout symptoms related to residents’ also improved from 2019 to 2022 with a small effect size of 0.201 (MD = 3.8). The category ‘Degrees of freedom (breaks/holidays)’ increased greatly with a small effect size of 0.455, and a mean difference between the two surveys of 9.6. ‘Presenteeism’, which refers to being present at work despite being ill, showed positive changes with a small effect size of 0.425 and a lower mean value of 11.8 points in 2022.

However, ‘Dissolution’, i.e., setting boundaries between work and private life, lead to higher psychosocial burdens in 2022 than in 2019, with small effect size of 0.217 and a mean difference of 5.4. Participants also perceived ‘Role conflicts’ as more burdensome in 2022 than in 2019 (d = 0.282; MD = 5.5) as well as ‘Role clarity’ (d = 0.251; MD = 3.3). Furthermore, ‘Burnout symptoms related to relatives’  increased from 2019 to 2022 with a small effect size of 0.318 and a mean difference of 6.0.

Although relevant changes of scales between 2019 and 2022 could be identified in eleven categories, only two of these changes were statistically significant according to the t-test, with a significance level of p < 0.05. ‘Degrees of freedom (breaks/holidays)’ improved from 2019 to 2022, with a statistically significant t-test result of -2.40 (p = 0.018). Results in the category ‘Presenteeism’ were also better in 2022 than in 2019, with a statistically significant t-test result of 2.26 (p = 0.026).

Table 2 shows the mean values and standard deviations of the COPSOQ scales. Furthermore, this table presents the mean differences and effect sizes of changes between 2019 and 2022 alongside the t-test values.

**Table 2:** Mean values and changes between 2019 and 2022

Category	Interpretation of high mean values	2019			2022			Effects			t-test^1^		
		n	M [95%-CI]	SD	n	M [95%-CI]	SD	MD	d^2^	+/-^3^	t-value	df	p-value
**Demands**													
Quantitative demands	negative	54	58.2 [52.71; 63.69]	20.6	57	52.3 [48.15; 56.45]	16.0	-5.9	0.321	+	1.68	99.98	0.096
Emotional demands	negative	55	76.6 [71.76; 81.44]	18.3	58	73.5 [68.87; 78.13]	18.0	-3.1	0.171		0.91	110.46	0.366
Hiding emotions	negative	55	48.0 [41.63; 54.37]	24.1	58	45 [38.98; 51.02]	23.4	-3.0	0.126		0.67	110.24	0.504
Work privacy conflicts^4^	negative	55	38.8 [30.92; 46.68]	29.8	58	38.9 [32.77; 45.03]	23.8	0.1	0.004		0.02	103.28	0.984
Dissolution	negative	55	37.5 [30.73; 44.27]	25.6	58	42.9 [36.65; 49.15]	24.3	5.4	0.217	-	-1.15	109.78	0.253
**Influence and possibilities for development**													
Influence at work	positive	55	48.0 [42.11; 53.89]	22.3	58	53.4 [47.74; 59.06]	22.0	5.4	0.244	+	-1.30	110.50	0.198
Degrees of freedom (breaks/holidays)	positive	55	59.8 [53.35; 66.25]	24.4	58	69.4 [64.92; 73.88]	17.4	9.6	0.455	+	-2.40	97.22	0.018
Possibilities for development	positive	55	66.4 [61.17; 71.63]	19.8	58	65.7 [60.78; 70.62]	19.1	-0.7	0.036		0.19	110.12	0.849
Meaning of work	positive	55	88.9 [83.06; 94.74]	22.1	58	89.0 [85.32; 92.68]	14.3	0.1	0.005		0.03	91.70	0.977
Commitment to workplace	positive	55	65.7 [58.06; 73.34]	28.9	58	69.8 [64.22; 75.38]	21.7	4.1	0.161		0.85	100.08	0.398
**Social relations and leadership**													
Predictability of work	positive	55	55.5 [49.18; 61.82]	23.9	58	59.5 [55.05; 63.95]	17.3	4.0	0.193		-1.02	98.06	0.313
Role clarity	positive	55	80.5 [76.85; 84.15]	13.8	58	77.2 [73.98; 80.42]	12.5	-3.3	0.251	-	1.33	108.50	0.186
Role conflicts	negative	55	38.8 [33.88; 43.72]	18.6	58	44.3 [39.08; 49.52]	20.3	5.5	0.282	-	-1.50	110.87	0.136
Quality of leadership	positive	55	61.7 [54.88; 68.52]	25.8	58	62.6 [56.91; 68.29]	22.1	0.9	0.038		-0.20	106.46	0.843
Support at work	positive	55	72.7 [67.18; 78.22]	20.9	58	75.2 [70.82; 79.58]	17.0	2.5	0.132		-0.70	104.17	0.488
Feedback	positive	55	57.3 [51.25; 63.35]	22.9	58	56.2 [50.74; 61.66]	21.2	-1.1	0.050		0.27	109.14	0.792
Quantity of social relations	positive	55	61.4 [54.24; 68.56]	27.1	58	56.9 [50.77; 63.03]	23.8	-4.5	0.177		0.94	107.43	0.351
Sense of community	positive	55	74.8 [70.41; 79.19]	16.6	58	77.4 [73.51; 81.29]	15.1	2.6	0.164		0.87	108.63	0.386
Unfair treatment	negative	55	24.5 [17.84; 31.16]	25.2	58	21.1 [15.34; 26.86]	22.4	-3.4	0.143		0.76	107.87	0.451
Trust and justice	positive	55	65.2 [61.00; 69.40]	15.9	58	67.3 [63.62; 70.98]	14.3	2.1	0.139		0.74	108.27	0.463
Recognition	positive	55	56.8 [49.24; 64.36]	28.6	58	61.6 [55.09; 68.11]	25.3	4.8	0.178		0.94	107.70	0.348
**Additional factors**													
Work environment/physical demands	negative	55	40.0 [34.24; 45.76]	21.8	58	42.1 [36.49; 47.71]	21.8	2.1	0.096		0.51	110.68	0.610
Job insecurity	negative	55	22.9 [17.85; 27.95]	19.1	58	16.4 [10.92; 21.88]	21.3	-6.5	0.321	+	1.71	110.66	0.091
Insecurity over working conditions	negative	55	29.1 [22.23; 35.97]	26.0	58	22.3 [17.26; 27.34]	19.6	-6.8	0.296	+	1.56	110.29	0.121
**Effects on job satisfaction and health**													
Intention to leave profession/job^4^	negative	55	14.5 [7.65; 21.35]	25.9	58	14.4 [9.18; 19.62]	20.3	-0.1	0.004		0.02	102.33	0.982
Job satisfaction	positive	55	65.2 [60.52; 69.88]	17.7	58	64.7 [60.92; 68.48]	14.7	-0.5	0.031		0.163	105.13	0.871
Work engagement	positive	55	73.8 [69.04; 78.56]	18.0	57	70.2 [65.22; 75.18]	19.2	-3.6	0.193		1.024	109.91	0.308
General health	positive	55	68.4 [62.56; 74.24]	22.1	57	71.9 [67.12; 76.68]	18.4	3.5	0.172		-0.91	105.07	0.365
Burnout symptoms	negative	55	52.3 [46.30; 58.30]	22.7	58	49.0 [43.78; 54.22]	20.3	-3.3	0.153		0.81	108.08	0.418
Presenteeism	negative	55	52.7 [45.54; 59.86]	27.1	58	40.9 [33.59; 48.21]	28.4	-11.8	0.425	+	2.26	111.00	0.026
Inability to relax	negative	53	43.4 [35.35; 51.45]	29.9	58	38.8 [31.85; 45.75]	27.0	-4.6	0.162		0.85	105.12	0.398
**Contact to residents and relatives**													
Conflicts with relatives	negative	55	15.0 [9.82; 20.18]	19.6	57	15.8 [12.01; 19.59]	14.6	0.8	0.046		-0.24	99.74	0.808
Burnout symptoms related to relatives	negative	55	26.0 [20.79; 31.21]	19.7	58	32.0 [27.34; 36.66]	18.1	6.0	0.318	-	-1.683	108.93	0.095
Conflicts with residents	negative	53	40.5 [34.20; 46.80]	23.4	58	39.1 [32.38; 45.82]	26.1	-1.4	0.056		0.298	108.96	0.766
Burnout symptoms related to residents	negative	53	31.0 [26.26; 35.74]	17.6	58	27.2 [22.03; 32.37]	20.1	-3.8	0.201	+	1.062	108.81	0.291

CI: Confidence interval; df: Degree of freedom; M: Mean; MD: Mean difference; n: Total number of individuals in the sample; SD: Standard deviation

^1^ Welch t-test (95%-CI, α = 0.05); ^2^ Cohen’s d (95%-CI); ^3^+: Relevant positive change between 2019 and 2022 (d ≥ 0.2); -: Relevant negative change between 2019 and 2022 (d ≥ 0.2); ^4^ Scale adjustments between 2019 and 2022

As the COVID-19-related modules employed in 2022 were not included in the 2019 survey, they were excluded from the comparison but are listed in detail in Table 3. The mean values of all items regarding ‘Organisation/communication’ during COVID-19 were in the upper third of the range, leading to a mean of 68.2 within this category. Items related to ‘Operational measures and overall assessment’ received ratings within or close to the upper quartile, resulting in a mean of 78.1.

**Table 3:** Single items of the two COVID-19 related modules

Category Items	Interpretation of high mean values	2022		
		n	M [95%-CI]	SD
**COVID-19: Organisation/communication**	**Positive**	**57**	**68.2** **[63.29; 73.11]**	**18.9**
The exchange/communication with my colleagues and managers is currently well.	positive	57	67.5 [62.02; 72.98]	21.1
I currently receive the support I need from my colleagues and managers to cope with present challenges.	positive	57	69.3 [63.82; 74.78]	21.1
I perceive the emotional support I receive from my work colleagues and managers as sufficient.	positive	57	65.8 [59.73; 71.87]	23.4
There is a good working atmosphere in my team/department despite the COVID-19 pandemic.	positive	57	70.2 [65.22; 75.18]	19.2
Did you work more hours than usual due to the COVID-19 pandemic?	negative	58	38.9 [31.31; 46.49]	29.5
**COVID-19: Operational measures and overall assessment**	**Positive**	**57**	**78.1** **[74.08; 82.12]**	**15.5**
I feel well informed by my company about the planned and implemented operational measures regarding the COVID-19 pandemic.	positive	57	76.3 [72.15; 80.45]	16.0
Due to the protective measures taken in our company, I feel well protected at my current workplace.	positive	57	76.8 [71.92; 81.68]	18.8
I consider the measures implemented or planned in our company with regard to the Corona virus as exaggerated.	negative	56	26.3 [17.97; 34.63]	31.8
I am afraid that I will bring the Corona virus home from work and thus endanger myself and my private environment.	negative	57	23.7 [16.30; 31.10]	28.5
Currently I am much more worried about my job than I was in the months before the COVID-19 pandemic broke out.	negative	56	11.6 [5.76; 17.44]	22.3

CI: Confidence interval; M: Mean; n: Total number of individuals in the sample; SD: Standard deviation

## Discussion

Positive and negative changes in psychosocial burdens were identified within this study. In the two COPSOQ surveys administered in 2019 and 2022, relevant changes in psychosocial burdens could be identified in eleven scales, in which context the four categories ‘Dissolution’, ‘Role conflicts’, ‘Role clarity’ and ‘Burnout symptoms related to relatives’ were perceived as worse in 2022 than in 2019. Positive changes from 2019 to 2022 were observed in the following seven categories: ‘Quantitative demands’, ‘Influence at work’, ‘Job insecurity’, ‘Insecurity over working conditions’, ‘Burnout symptoms related to residents’, ‘Degrees of freedom (breaks/holidays)’ and ‘Presenteeism’. However, only the changes in ‘Degrees of freedom (breaks/holidays)’ and ‘Presenteeism’ were shown to be statistically significant by the t-test. The COVID-19-related modules ‘Organisation/communication’ and ‘Operational measures and overall assessment’ were rated in the upper third and upper quartile, respectively.

No study specifically addressing the psychosocial burdens of palliative care among nursing staff working in German nursing homes and the ways in which these burdens were affected by the COVID-19 pandemic could be found. However, six studies that analysed the impact of the pandemic on psychosocial burdens in German nursing homes in general were identified [14, 17–21], two of them which also used the COPSOQ questionnaire [17, 19]. However, COVID-19 exposure and experiences may have varied due to the focus on different regions in Germany, as the federal states implemented different COVID-19 measures over time. Furthermore, the studies were conducted in different periods during the pandemic; thus, experiences of psychosocial burdens may have varied among these periods. Although individuality must be taken into account when putting these results into perspective, similarities to other studies may indicate common tendencies.

As part of a cross-sectional study, Schulze et al. compared the COPSOQ results reported by their study sample to a German pre-pandemic reference group consisting of geriatric nurses from the COPSOQ database [17]. Consistent with the results of this study, they found negative changes in the category ‘Role conflicts’ [17]. In other studies, staff working at nursing homes reported higher work demands due to isolation and lockdowns, which not only changed their role as they tried to compensate residents’ families, social workers and other visitors in terms of social contact but also caused them to feel compassion for residents, leading to guilty consciences whenever the lack of time resources prevented them from providing adequate care to residents [18, 21]. This situation might have led to role conflicts as a possible explanation of the relevant negative changes within this category according to the present study.

Another inner conflict that may extend beyond working hours and could resonate with the negative changes associated with ‘Dissolution’ might be to the result of perceived grief, stress and anger as well as sleeping disorders due to the pandemic [20, 21]. Diehl et al. further identified severe psychological stress during the pandemic resulting from the fear of becoming infected [18], which has also been reported in other studies [20, 21]; however, this important aspect could not be confirmed by reference to the COVID-19-related COPSOQ items included in this study, as the majority of participants answered that they felt well protected at work and indicated that they were unafraid of becoming infected at work and subsequently infecting their private environments (see Table 3).

While Schulze et al. identified negative changes in ‘Burnout symptoms’ in general [17], the results of the present study only indicated relevant negative changes in ‘Burnout symptoms related to relatives’ (see Table 2). In addition to more difficulties in communication with relatives, other studies have also identified more conflicts with residents [18], besides relatives, due to contact restriction as well as the failure to respect hygiene regulations and guidelines [18, 21]. However, contrary to this, participants of the present study perceived less burnout symptoms related to residents in the survey before the COVID-19 pandemic. 

In the sample studied by Schulze et al., ‘Presenteeism’ underwent negative changes during the pandemic [17], while this category exhibited a relevant and statistically significant improvement in the sample used in this study (see Table 2). Increased awareness of one’s own symptoms of sickness as well as the fear of infecting others [18, 20, 21], especially residents who could suffer serious consequences, might lead to a greater sense of responsibility with regard to the staff’s decision not to come to work when having signs of illness. This situation could result in less presenteeism and explain the relevant and statistically significant findings of the present study. On the other hand, the sample investigated by Schulze et al. might have worked under different organisational and personnel-related working conditions, thus rendering making sick leave impossible during outbreaks. However, these different results regarding the category ‘Presenteeism’ remain unclear.

Schulze et al. identified further negative changes that occurred during the pandemic in the categories ‘Work privacy conflicts’, ‘Quality of leadership’, ‘Quantity of social relations’, ‘Work environment’, ‘Intention to leave profession/job (past 12 Months)’ and ‘Inability to relax’ as well as positive changes in the category ‘Recognition’ [17], which were unremarkable in this sample. However, as no pre-pandemic COPSOQ data pertaining to the same type of sample were available, comparison and evaluation of the pandemic effects were limited.

Diehl et al. conducted a qualitative study by interviewing nurses who worked in nursing homes; these nurses mentioned intensified care work and additional pandemic-related tasks leading to time constraints [18]. Additional COVID-19-related work tasks as well as staff absences resulting in higher psychosocial and physical strains have also been identified in other studies [14, 20, 21]. The interviewees in Diehl et al.’s study furthermore perceived the fact that they worked under conditions of frequently changing guidelines and regulations, which resulted in constantly changing working processes, as stressful [18]. Controversy, in this study, ‘Quantitative demands’ and ‘Insecurity over working conditions’, which focus on relatable topics, exhibited relevant positive changes during the pandemic. As autonomy in nursing practice has been identified as an important aspect of work motivation and personal resilience [40], the more highly rated categories ‘Influence at work’ (relevant) and ‘Degrees of freedom (breaks/holidays)’ (relevant + statistically significant) could be related to gains in personal resources and thus to more positive perception of quantitative demands and less insecurity over working conditions. Additionally, the rather positive perceptions of organisational working conditions during the pandemic revealed by the COVID-19-related COPSOQ scales (see Table 3) might contribute to this situation.

Until May 2020, 49% of all COVID-19 deaths in Germany occurred among nursing home residents [41]. Additionally, the interviewees in the study conducted by Diehl et al. mentioned experiencing far more deaths among residents than usual, especially during outbreaks, a situation which was perceived as physically difficult and emotionally frustrating [18]. However, the results of the present study indicated no differences in ‘Emotional demands’, which might indicate milder or less severe COVID-19 outbreaks within the facilities surveyed and/or a lack of effects on mortality among residents. Another possible reason why the results in this study did not indicate higher ‘Emotional demands’ during the COVID-19 pandemic might be due to the resilience of participants. A recent scoping review identified meaning in work and strong team and supervisor relationships as important resources for addressing challenges during COVID-19 outbreaks [42]. Additionally, Hering et al. 2022, who used three COPSOQ subscales to analyse the associations between the characteristics of nurses and psychosocial burdens, found significant associations between stress and ‘Support at work’ as well as ‘Sense of community’ [19]. Furthermore, a sense of duty, pride due to being a member of a caring profession during such a difficult time and collective peer support were identified that strengthened personal resources which helped with coping [43, 44]. Positive tendencies within the categories ‘Meaning of work’, ‘Commitment to workplace’, ‘Recognition’, ‘Quality in leadership’, ‘Support at work’, ‘Sense of community’ and ‘Trust and justice’ (see Table 2) as well as rather positive perceptions of COVID-19-related measures, working conditions and support from the organisation (see Table 3) might indicate sources of resilience, that can help staff cope with a multitude of emotional demands.

### Limitations

This study aims to provide further insights into the impact of the pandemic on the psychosocial burdens of palliative care among nursing staff of nursing homes in Germany. Pandemic-related changes in psychosocial burdens could be identified using a pre-post-comparison.

However, changes in psychosocial burdens between 2019 and 2022 could only be identified, but not explained as the COPSOQ-survey does not indicate the reasons for such changes. Therefore, the question of why ‘Quantitative demands’ or ‘Presenteeism’, for example, were perceived as more positive during the pandemic according to this study while ‘Role conflicts’ increased, remains unanswered. Organisational and employment information that could offer more insights into the possible reasons of these impacts were not explored within the scope of the survey and may have influenced the results. Furthermore, the study sample included nurses, nursing assistants and caregivers, which represent the professions that are most involved in the provision of palliative care in nursing homes. However, it would be interesting to analyse the differences between the responses provided by these groups, which was not the focus of this study due to its small number of participants.

Moreover, data were collected in the context of palliative care, and participants were informed both in writing and verbally to report on their experiences in palliative care during their everyday work in the nursing homes. However, as palliative care is part of their work alongside other tasks associated with general care practices, it possibility that general psychosocial burdens at work of the respondents besides palliative care have been incorporated within the answers of the standardized COPSOQ-scales cannot be excluded. Due to its sector-specific design, reliability and validity as well as its internationally broad scope application, the COPSOQ was still considered to be the most appropriate instrument for this research. However, the fact that the reported reliability and homogeneity of the methods refer to the standard version and does not include the additional COPSOQ modules must be noted. This point should be taken into account when discussing or comparing of the study results with those of other studies focusing on pandemic-related psychosocial burdens in German nursing homes in general.

### Generalisability

Aspects that affected workload and burdens in German nursing homes during the pandemic have been identified by several studies [14, 17–21], but these results may not be in line with this sample, especially since this study focused on palliative care. Furthermore, this study was conducted in North Rhine-Westphalia, Germany, while the protection rules implemented during the pandemic differed across the federal states, which may have led to different impacts on psychosocial burdens. Furthermore, nursing homes may not have been equally affected by COVID-19, which may have led to bias.

Another point that may affect the generalisability of these results is that the question of how other aspects, such as staff turnover and changes in legal requirements in the time between the two survey periods, may have influenced responses and thus led to bias remains unanswered. With regard to the second survey period, data collection was performed at a time when it was once again acceptable to conduct a survey of the workforce; accordingly, the peak of the COVID-19 pandemic had already passed. One month prior to the survey period, coronavirus restrictions had been greatly relaxed, and it cannot be determined how the prospect of such a light at the end of the tunnel may have led to bias and to more positive assessments.

Furthermore, the results of this study are limited due to its relatively small number of participants, who were drawn from only two nursing homes, which nevertheless offered the opportunity to perform a pre-post-comparison to analyse the impacts of the pandemic on psychosocial burdens. This rare opportunity for such a pre-post-comparison enabled to provide exclusive insights into this unexplored research topic. However, further research is necessary to assess generalisability of this research more effectively, address possible selection bias and obtain further insights into this field of research that can be contrasted to the results of the present study.

## Conclusions

During the COVID-19 pandemic, German nursing homes and their staff faced immense pressure, thus adding further burdens to their already precarious working conditions. However, in addition to negative changes, some scales were assessed as more positive during the second survey period. Furthermore, the organisation/communication and operational measures and the overall assessment during the COVID-19 pandemic were perceived as rather positive according to the results of this study, indicating that perceptions of challenges in palliative care in nursing homes during the pandemic were highly dependent on organisational working conditions and support that can strengthen the individual resources and resilience of the workforce. Hence, a greater focus on the establishment of supportive organisational working conditions, the enhancement team and supervisor relationships, and the provision of recognition and appreciation, as important sources of resilience, could support the attempts of the individual and thus the team to cope with the multitude of work demands associated with palliative care and their professions.

### Electronic supplementary material

Below is the link to the electronic supplementary material.

Supplementary Material 1

## Data Availability

The datasets supporting the conclusions of this article are included within the article.
