# Supplementary material for: Psychosocial burdens in palliative care – a longitudinal cohort study in nursing homes and impacts of the COVID-19 pandemic
Source: BMC Palliat Care. 2023 Oct 28;22:163. doi: 10.1186/s12904-023-01292-4 (PMC10612279; doi:10.1186/s12904-023-01292-4)
Supplement: Supplementary file 1 — Supplementary Material 1 [file 12904_2023_1292_MOESM1_ESM.pdf]

Supplementary material

Hartley's Fmax Test

Category	2019				2022				Fmax[1]	
	n	M	SD		n	M	SD		F	p-value
**Demands**										
Quantitative demands	54	58.2	20.6		57	52.3	16.0		1.658	0.031
Emotional demands	55	76.6	18.3		58	73.5	18.0		1.034	0.450
Hiding emotions	55	48	24.1		58	45	23.4		1.061	0.412
Work privacy conflicts[2]	55	38.8	29.8		58	38.9	23.8		1.568	0.048
Dissolution	55	37.5	25.6		58	42.9	24.3		1.110	0.347
**Influence and possibilities for development**										
Influence at work	55	48.0	22.3		58	53.4	22.0		1.027	0.459
Degrees of freedom (breaks/holidays)	55	59.8	24.4		58	69.4	17.4		1.966	0.006
Possibilities for development	55	66.4	19.8		58	65.7	19.1		1.075	0.393
Meaning of work	55	88.9	22.1		58	89.0	14.3		2.388	0.001
Commitment to workplace	55	65.7	28.9		58	69.8	21.7		1.774	0.016
**Social relations and leadership**										
Predictability of work	55	55.5	23.9		58	59.5	17.3		1.909	0.008
Role clarity	55	80.5	13.8		58	77.2	12.5		1.219	0.229
Role conflicts	55	38.8	18.6		58	44.3	20.3		1.191	0.258
Quality of leadership	55	61.7	25.8		58	62.6	22.1		1.363	0.123
Support at work	55	72.7	20.9		58	75.2	17.0		1.511	0.061
Feedback	55	57.3	22.9		58	56.2	21.2		1.167	0.281
Quantity of social relations	55	61.4	27.1		58	56.9	23.8		1.297	0.165
Sense of community	55	74.8	16.6		58	77.4	15.1		1.209	0.239
Unfair treatment	55	24.5	25.2		58	21.1	22.4		1.266	0.189
Trust and justice	55	65.2	15.9		58	67.3	14.3		1.236	0.213
Recognition	55	56.8	28.6		58	61.6	25.3		1.278	0.179
**Additional factors**										
Work environment/physical demands	55	40.0	21.8		58	42.1	21.8		1.000	0.499
Job insecurity	55	22.9	19.1		58	16.4	21.3		1.244	0.209
Insecurity over working conditions	55	29.1	26.0		58	22.3	19.6		1.760	0.018
**Effects on job satisfaction and health**										
Intention to leave profession/job[2]	55	14.5	25.9		58	14.4	20.3		1.628	0.036
Job satisfaction	55	65.2	17.7		58	64.7	14.7		1.450	0.082
Work engagement	55	73.8	18.0		57	70.2	19.2		0.879	0.316
General health	55	68.4	22.1		57	71.9	18.4		1.443	0.086
Burnout symptoms	55	52.3	22.7		58	49.0	20.3		1.250	0.201
Presenteeism	55	52.7	27.1		58	40.9	28.4		1.098	0.364
Inability to relax	53	43.4	29.9		58	38.8	27.0		1.226	0.224
**Contact to residents and relatives**										
Conflicts with relatives	55	15.0	19.6		57	15.8	14.6		1.802	0.015
Burnout symptoms related to relatives	55	26.0	19.7		58	32.0	18.1		1.185	0.262
Conflicts with residents	53	40.5	23.4		58	39.1	26.1		1.244	0.211
Burnout symptoms related to residents	53	31.0	17.6		58	27.2	20.1		1.304	0.164

[1] Hartley's Fmax Test to test for homogeneity of variances (95 %-CI, α=0.05); [2] Scale adjustments between 2019 and 2022
M: Mean; MD: Mean difference; n: Total number of individuals in the sample; SD: Standard deviation.
